# Recurrent Large Bowel Obstruction Caused by Necrotizing Pancreatitis: A Rare Manifestation

**DOI:** 10.7759/cureus.12307

**Published:** 2020-12-26

**Authors:** Mehak Bassi, Anjali Desai, C.S. Pitchumoni

**Affiliations:** 1 Internal Medicine, Saint Peter's University Hospital, New Brunswick, USA; 2 Medicine, Robert Wood Johnson University Hospital, New Brunswick, USA; 3 Gastroenterology and Hepatology, Saint Peter’s University Hospital, New Brunswick, USA

**Keywords:** acute necrotizing pancreatitis, acute pancreatitis complications, recurrent large bowel obstruction, walled-off pancreatic necrosis

## Abstract

Mechanical obstruction of the colon is rare with necrotizing pancreatitis but is associated with high morbidity and mortality. However, pancreatic ileus, colonic necrosis, and pancreatic colonic fistulae with necrotizing pancreatitis are well known. The anatomic proximity of the pancreas to the transverse colon becomes clinically relevant when a patient with pancreatitis demonstrates a localized ileus of the transverse colon (an old term “the colon cut-off sign”), even when the disease is mild, or lower gastrointestinal bleeding secondary to necrosis of the segment in severe acute pancreatitis. We present the case of a 25-year-old female with choledocholithiasis who presented with severe abdominal pain and was found to have recurrent large bowel obstruction secondary to walled-off pancreatic necrosis. Bowel obstruction is a rare complication of walled-off necrosis, but clinicians should be aware of it due to significantly increased mortality rates. Recurrent bowel obstructions are rarely known in necrotizing pancreatitis and may warrant a bowel resection either electively or acutely. Walled-off necrosis does not respond to typical treatment of symptomatic pseudocysts, which includes endoscopic cystogastrostomy or percutaneous drainage with small-bore catheters. Endoscopic or surgical necrosectomy is necessary for the resolution of walled-off necrosis to evacuate the non-liquefied components.

## Introduction

Acute necrotizing pancreatitis is rarely known to cause life-threatening colonic necrosis, bleeding, or perforation while eroding the large bowel. Colonic involvement is usually difficult to diagnose both radiologically and clinically. Walled-off necrosis is a complication of necrotizing pancreatitis. Recurrent bowel obstruction secondary to walled-off necrosis is extremely rare. The underlying pathology is due to anatomic proximity, which leads to the contiguous spread of inflammatory cascade between the pancreas and the colon. It is vital for the clinician to know this because non-specific symptoms and systemic characteristics of a critical illness make it hard to acknowledge that the large bowel may be involved. Colonic resection is commonly required to treat severe cases of large bowel obstruction (LBO) that are caused by acute necrotizing pancreatitis [1].

## Case presentation

**Figure 1:**
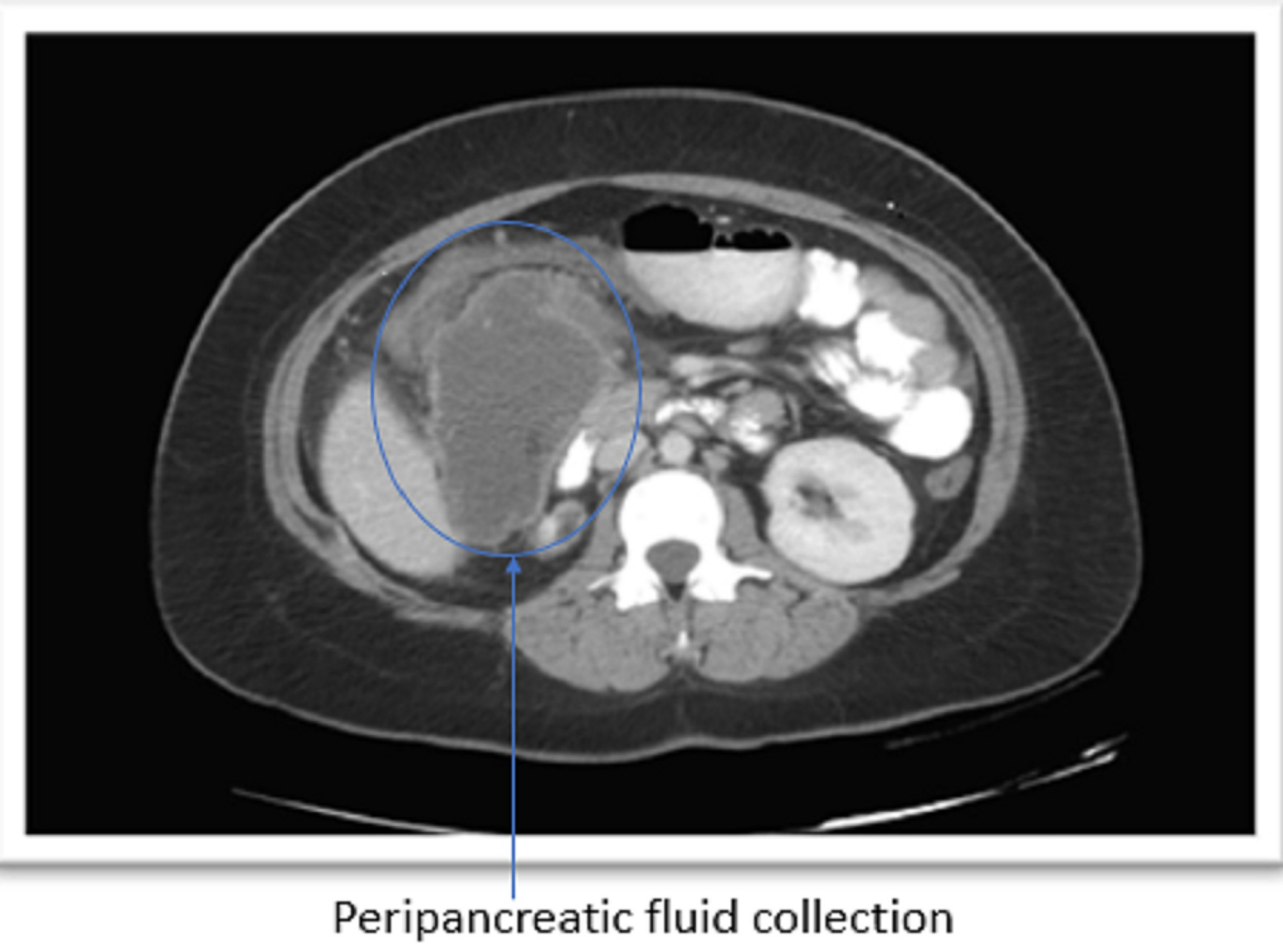
Coronal CT of the abdomen section at the level of pancreas confirming a large 7.7 x 8.8 cm rim-enhancing fluid collection.

A 25-year-old Mexican female was doing well until two days ago before presenting to the hospital with diffuse abdominal pain, nausea, and multiple episodes of bilious non-bloody vomiting. Her last bowel movement was two days prior to coming to the hospital. One month prior, she was admitted for choledocholithiasis. She was managed then with endoscopic retrograde cholangiopancreatography (ERCP), and a stent was placed during sphincterotomy. At the time, her hospitalization was complicated by post-ERCP pancreatitis. The patient recovered two days later from conservative management. In the ED, the patient was noted to be diaphoretic and anxious. Physical examination showed tachycardia and a distended abdomen with hypoactive bowel sounds and diffusely moderate tenderness on palpation. Basic laboratory testing, including lipase, was unremarkable. An urgent CT scan confirmed the presence of a large 7.7 x 8.8 cm rim-enhancing fluid collection, described initially as a pseudocyst (Figure 1).

**Figure 2:**
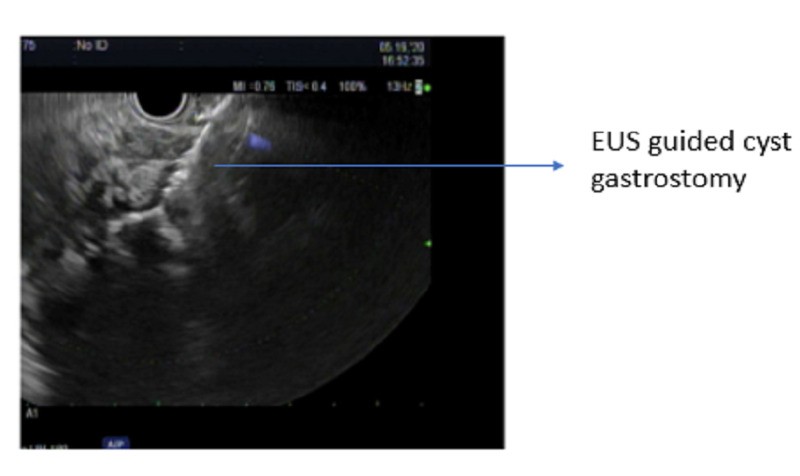
Endoscopic ultrasound guided cystogastrostomy.

**Figure 3:**
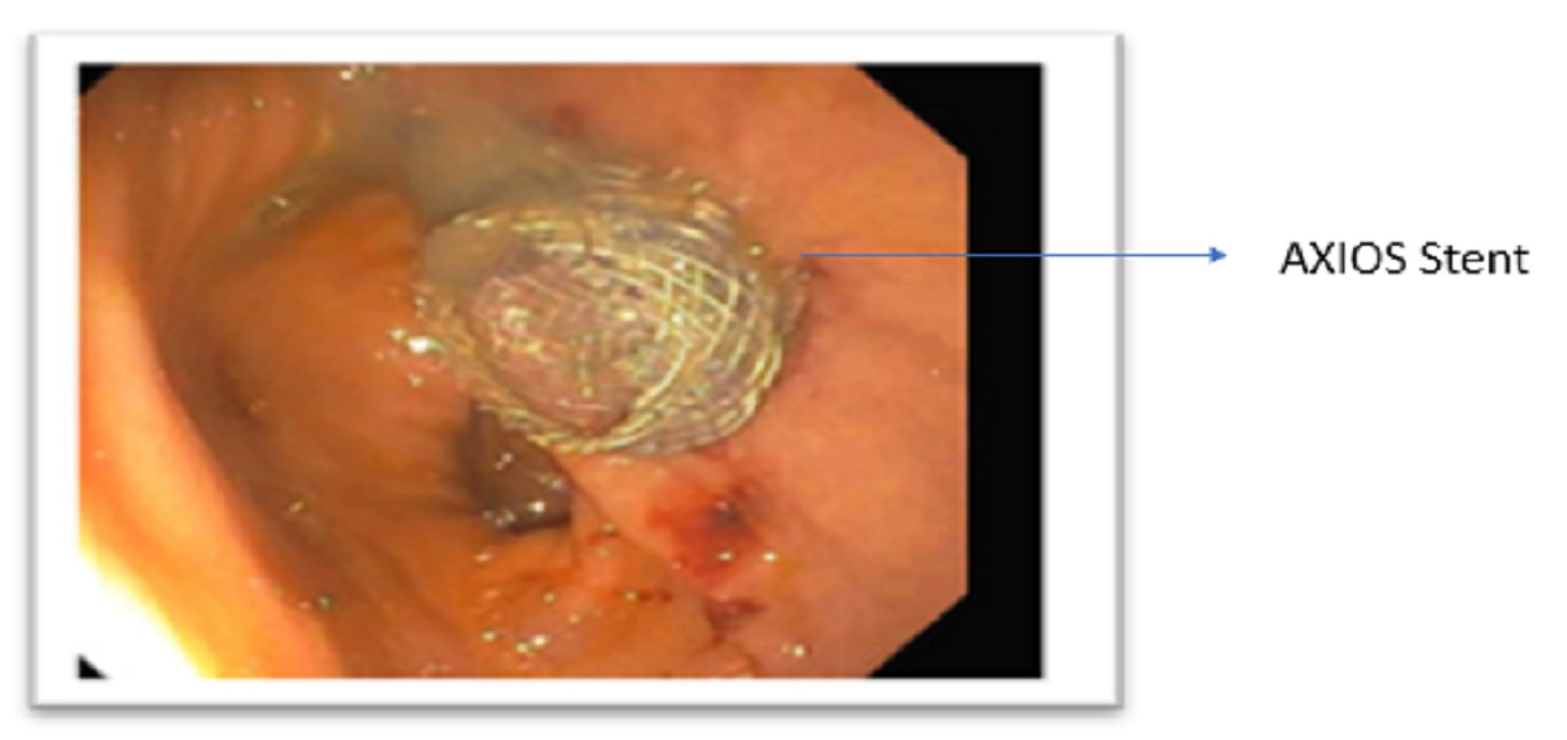
AXIOS stent placement during cystogastrostomy.

There was an interval improvement of the previously seen peri-pancreatic edema. Remarkably, there was a colonic transition point near the hepatic flexure regional to the peri-pancreatic collection with decompression of the distal colon. The ascending colon was dilated and fluid-filled with admixed contrast. The findings were consistent with LBO and secondary partial small bowel obstruction (SBO) caused by extrinsic compression of the hepatic flexure by the pancreatic collection. A nasogastric tube was placed for immediate decompression. The patient underwent an endoscopic ultrasound guided cystogastrostomy (Figure 2) using the AXIOSä stent system (Boston Scientific, Marlborough, MA, USA) (Figure 3).

**Figure 4:**
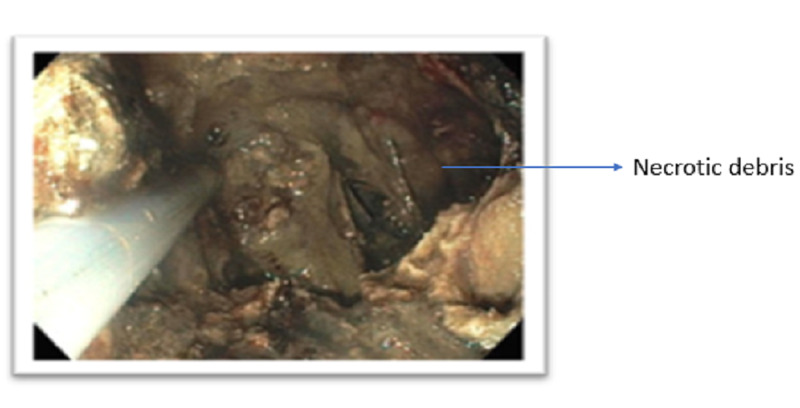
Necrotic debris indicating walled-off necrosis.

The cyst contained necrotic debris, most consistent with walled-off necrosis (Figure 4).

**Figure 5:**
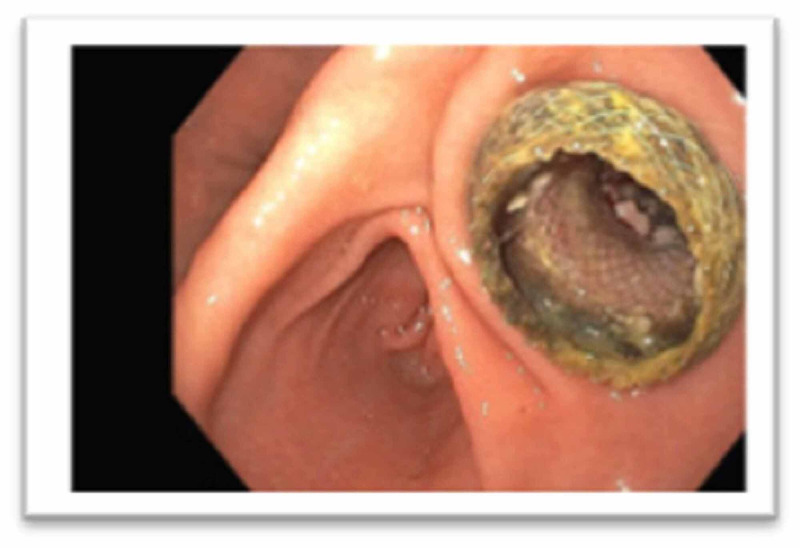
Residual debris after repeat necrosectomy.

The following day, the patient had mild improvement in her symptoms but was still unable to pass bowel movements. A repeat CT scan showed a mild decrease in the size of the pancreatic cyst but no significant improvement in LBO/SBO. The patient underwent EGD for necrosectomy of the walled-off necrosis. The patient improved clinically over the next two days, with improvement in abdominal pain along with the resumption of bowel movements, and was eventually discharged home. One month later, the patient underwent an elective EGD for stent removal and repeat necrosectomy for residual debris (Figure 5).

The previously seen pancreatic collection was significantly smaller on endoscopic examination. Another month later, she presented to the ED with complaints of abdominal pain and vomiting for one day. A CT scan showed a dilated ascending colon through the hepatic flexure with the distal transverse and collapse of the descending colon, which was suggestive of partial obstruction at the hepatic flexure. The resolving walled-off necrosis measured 5.7 cm x 2.1 cm. Given the above findings, she was admitted for an LBO secondary to inflammatory changes from the pancreatic walled-off necrosis. The patient responded to conservative management. Given the refractory LBO over the last two months, once the patient could tolerate diet, she underwent a colonoscopy to rule out any intraluminal etiologies. An extrinsic moderate stenosis was found at the hepatic flexure and was transverse, and colonic biopsies were normal. She was eventually discharged uneventfully with full resolution of her symptoms and has remained asymptomatic thereafter.

## Discussion

Our case highlights the importance of unusual complications of walled-off necrosis leading to LBO. Our patient presented with non-specific symptoms of abdominal pain and vomiting. Although the CT scan showed the possibility of the pancreatic pseudocyst, the endoscopic picture was suggestive of necrosis. Almost 30 case reports have demonstrated LBO in the setting of pancreatitis, both through the direct mass effect of the cyst and through extrinsic inflammatory changes [2]. Walled-off necrosis is a late complication of necrotizing pancreatitis (>four weeks), which originates from acute necrotic collections. It has well-defined, non-epithelialized walls and contains a necrotic mixture of fat and fluids from both pancreatic and peri-pancreatic tissue. Diagnosis is more succinct when a pancreatic collection increases in size, has a presence of fat attenuation debris, has an irregular border, and extends into the paracolic gutter [3]. Walled-off necrosis occurs in less than 10% of acute pancreatitis cases and within four to six weeks after the initial episode. The mortality rate is estimated to be between 10% and 30%. Approximately 30% of patients with pancreatic necrosis develop infected necrosis, and mortality can reach 100% if left untreated. The prevalence of colonic invasion is rare; however, the consequence is fatal, with mortality above 50% [4]. The most common site of accumulation of pancreatic inflammatory fluid is the lesser sac limited anteriorly by the stomach, laterally by the spleen, splenic flexure on the left, duodenum on the right, and inferiorly by the transverse mesocolon. The underlying pathophysiology relates to the direct extension of the inflammatory cascade, which may spread to the large bowel due to anatomic contiguity of the pancreas with the transverse and descending colon [5].

The first case of colonic obstruction was reported by Fortini. Intestinal obstruction due to pancreatitis can be of mechanical, spasmodic, or paralytic type. The well-known “colon cut-off sign” is a result of a visceral reflex stimulated from the superior mesenteric plexus [6]. Mechanical obstruction due to adjacent compression and displacement of organs is possible by inflammatory changes and the development of a collection through a necrotizing pancreatitis process. In studies conducted in the past, it has been found that colonic involvement is an unlikely obstacle that necrotizing pancreatitis may pose. Among all cases of acute pancreatitis, necrosis of the large bowel happens in 1% of them. Its incidence in necrotizing pancreatitis has been found to be between 6% and 40% [7]. Inflammatory changes may cause adjacent fat stranding, mural hyperenhancement, and bowel wall thickening [8]. Walled-off necrosis does not respond to typical treatment of symptomatic pseudocysts, which include endoscopic cystogastrostomy or percutaneous drainage with small-bore (10-F or smaller) catheters. Endoscopic or surgical necrosectomy is necessary for the resolution of walled-off necrosis to evacuate the non-liquefied components [2]. In the past, open necrosectomy was considered as the definitive treatment; however, it is associated with the need for repeat intervention in up to 16% of cases and up to 92% post-procedure-related adverse events [9]. Less invasive procedures such as endoscopic necrosectomy (through the cystogastrostomy) have become more popular in recent times, resulting in shorter hospital stay, relatively shorter procedural time, and lower peri-procedural morbidity [10]. Moreover, patients who are not surgical candidates can undergo these minimally invasive procedures, thus increasing the therapeutic efficacy [11]. Recent studies have shown successful drainage and resolution in 93% of cases, with a complication rate of 14% [12].

## Conclusions

We reported a case where recurrent LBO was caused by walled-off necrosis, which is a rare occurrence. There have not been many such known cases of recurrent LBO from walled-off necrosis. We reported this case to alert the clinicians about pitfalls in diagnosis and potential long-term complications of necrotizing pancreatitis causing recurrent LBO. Often, colonic complications go unnoticed due to non-specific symptoms such as abdominal pain. Clinicians should strongly consider the involvement of colonic complications in patients who are not improving with aggressive medical therapy in pancreatitis. Often, these patients need regular follow-ups for the subsequent treatment of walled-off necrosis. Endoscopic or surgical necrosectomy is necessary for resolution and is preferred over open necrosectomy.
